# Predictors of Satisfaction in Patients with Knee Osteoarthritis Treated with a Single Injection of Mannitol-Modified Crosslinked Hyaluronate Derivative

**DOI:** 10.3390/jcm13185372

**Published:** 2024-09-11

**Authors:** Martin Balblanc, Anne Lohse, Frederic Meyer, Charles Rapp, Charlotte Bourgoin, Jean-Charles Balblanc, Thierry Conrozier

**Affiliations:** 1 General Medicine, Paris-Saclay University, 90014 Le Kremelin-Bicêtre, France; mbalblanc@gmail.com; 2 Department of Rheumatology, Hôpital Nord Franche-Comté, 90015 Belfort, France; anne.lohse@hnfc.fr (A.L.); cj-rapp@outlook.fr (C.R.); jean-charles.balblanc@hnfc.fr (J.-C.B.); 3 Clinical Research Unit, Hôpital Nord Franche-Comté, 90014 Belfort, France; charlotte.bourgoin@hnfc.fr

**Keywords:** satisfaction, patient opinions, hyaluronic acid, viscosupplementation, knee, osteoarthritis, effectiveness, predictors

## Abstract

**Background/Objectives:** There is a gap between the very positive opinion of patients and doctors regarding knee viscosupplementation (VS) and the contrasting results of controlled studies. The objective of this study was to evaluate the overall satisfaction and predictors of satisfaction with VS in patients with knee osteoarthritis treated with VS. **Methods**: Post-hoc analysis of a cross-sectional study in patients with knee OA treated with one injection of a mannitol-modified cross-linked HA (HANOX-M-XL). The primary outcome was satisfaction, self-assessed semi-quantitatively by the patients. Demographics, radiological features, comorbidities, OA and comorbidities treatments, and lifestyle associated with satisfaction were studied in bivariate and multivariate analysis. **Results**: 89 patients (124 knees) were analyzed. A total of 88.7% were satisfied with the treatment. Satisfaction was correlated with duration of effectiveness (DoE) and negatively correlated with BMI. Satisfaction was higher in active versus sedentary patients, in tibiofemoral involvement, in Kellgren-Lawrence grade 1–3 versus 4, and in subjects not requiring intraarticular corticosteroid (IACS) concomitantly to VS. Satisfied subjects were older than dissatisfied ones. In multivariate analysis, older age, K–L grade < 4, absence of IACS, and longer DoE were associated with higher rates of satisfaction. **Conclusions**: We identified several predictive factors of patient satisfaction after VS of the knee. Alongside these objective factors, there are probably subjective factors linked to patient beliefs, fears, and expectations impacting satisfaction.

## 1. Introduction

Satisfaction is a feeling of contentment and fulfillment that occurs when a person’s needs or expectations are met. Treatment satisfaction refers to the patient’s overall satisfaction with the medical care they received, including various factors such as the perceived effectiveness of the treatment, the level of symptom relief, the adequate response to expectations, fears and beliefs, with the consequence of improving the quality of life. Knee osteoarthritis (OA) is one of the leading causes of pain and disability in subjects above the age of 50, which can lead, in certain advanced cases, to severe impairment of quality of life [1]. The incidence of OA of the knee is influenced by numerous factors [2] with aging at the forefront [3]. Overweight and obesity considerably increase the load on joints, accelerating their degeneration. Recent research highlights metabolic and immune dysregulation in obese patients and underlines the role of dyslipidemia and insulin resistance in low-grade systemic inflammation leading to OA, via the release of various adipokines [4]. Severe injuries [5] (i.e., anterior cruciate ligament rupture, meniscus tear…) and biomechanical abnormalities (i.e., varus or valgus malalignment, trochlea dysplasia) leading to increased tibiofemoral contact stress [6] can also result in the development of this condition. Both lack of physical activity and excessive joint strain (as seen in professional athletes) affect joint health. Additionally, certain comorbidities, such as rheumatoid arthritis, can elevate the risk of OA. Lifestyle factors (i.e occupational and work activities) as well as a diet deficient in essential nutrients also contribute to the likelihood of developing OA [7]. Lastly, genetics plays a significant role, potentially predisposing individuals to OA [8].

Knee OA conservative treatments must include a combination of non-pharmacological and pharmacological methods [9,10,11,12], since each of them, taken separately, has only mild to moderate effectiveness [13]. Although it also can affect younger subjects, knee OA mainly affects elderly and frail patients who are, therefore, exposed to the more adverse effects of treatments as well as drug interactions [14]. Furthermore, in knee OA, as in all chronic pathologies, proper application of the therapeutic recommendations is a key element in success of the treatment [15]. For these reasons, the development of therapeutic strategies based on safe and well-tolerated drugs, injected into the joint once or twice a year, must be favored [16]. Corticosteroids are mainly indicated in patients with OA flares-ups [9,17] whose main characteristic is the presence of joint effusion. In periods when flare-ups are not present, viscosupplementation (VS) by hyaluronic acid (HA) intra-articular (IA) injection is preferred with the aim of decreasing pain and improving knee joint function for a long period of time [18]. VS is recommended by numerous learned societies [9,10,11,12,16,17,18,19], but only under certain conditions for some of them [20]. Only a few societies do not recommend the use of HA injections, basing their recommendations on a level of evidence assessed as insufficient [21]. However, there is a gap between these guidelines and clinical daily practice since, despite negative or lukewarm recommendations, more and more practitioners worldwide are continuing to use VS with satisfying clinical results [22], despite VS being primarily indicated in mild to moderate OA, where it has been shown to be most effective [23,24]. IA-HA can be useful for advanced disease [25,26], allowing a substantial number of patients to delay the need for knee replacement [27,28,29]. Several VS dosing regimens are currently available, depending on the preference of physicians and patients. Repeat injections of linear HA are gradually replacing single injections of long-lasting HA. The best validated way to increase the IA residence time of HA is to cross-link HA linear macromolecules using cross-linking agents [30]. HANOX-M-XL (HappyCross^®^, LABRHA SAS, Lyon, France) is an extended-release hyaluronic acid derivative, made of highly cross-linked HA combined with mannitol, a powerful antioxidant that protects HA from the reactive oxygen species (ROS) related depolymerization [31,32]. Thanks to its long-lasting effectiveness [33] due to a high resistance to ROS degradation [34], it is permitted for HANOX-M-XL to be administered through a single injection regimen. There are two main reasons why patients choose a long-acting viscosupplement, despite its higher cost. First, an IA injection is an invasive and moderately painful procedure that many patients want to space out as much as possible. Finally, it is increasingly difficult to find a practitioner trained in this type of injection, particularly in France where the density of rheumatologists is low (3.8 per 100,000 inhabitants in 2022) [35]. In 2021 and 2022, 83% of the patients referred to our rheumatology department for knee VS opted for a single injection rather than three. We follow advice of the Eurovisco group which recommends that, before making a therapeutic decision, caregivers must carefully consider the patient’s needs (i.e., pain relief, recreational or competitive sports practice, postponement of surgical intervention), their fears (permanent disability, surgical intervention, unpleasant side effects linked to treatments, etc.), as well as their preferences and expectations [16]. Due to different patient expectations, satisfaction with a treatment can vary significantly. Even though, in daily clinical practice, patient opinions are essential for evaluating the effectiveness of OA treatments, satisfaction with treatment is a patient-reported outcome (PRO) that is rarely considered in controlled studies. In such studies, effectiveness is evaluated mainly on the variation of composite scores, which is both complex and time-consuming, thereby making them difficult to use in daily medical practice. For Migliore et al., this may explain the gap between the very positive impression of patients and doctors regarding VS and the more contrasting results of published studies [22].

The primary objective of this study was to evaluate the overall satisfaction of patients with knee OA treated with a single IA injection of HANOX-M-XL. The second objective was to identify patient characteristics that accurately predict whether patients will be satisfied or dissatisfied, to help practitioners select those who are most likely to be satisfied with treatment.

## 2. Patients and Methods

This work is a *post-hoc* analysis of a cross-sectional, single-center trial (PRESAGE Study; ClinicalTrials.gov Identifier: NCT04988698), carried out under conditions of daily clinical practice, primarily designed to assess the predictive factors of the duration of effectiveness of VS in knee OA patients [36]. The trial received the approval of the French “Comité de Protection des Personnes Sud EST III” under ID-CRB N° 2021-A00773-38 and was conducted in accordance with good clinical practices and the Declaration of Helsinki.

### 2.1. Patient Selection

All the ambulatory adult patients, female or male, regardless of age, who were referred to the rheumatology department of the North Franche-Comté Hospital (Belfort, France) more than 2 months and less than 3 years after being treated with VS, for symptomatic knee OA, were prospectively included in the study. In this report, only those treated with HANOX-M-XL, one injection of 2.2 mL, were studied. Patients who were unable to understand the questions due to cognitive problems or language barriers as well as those who did not accept to give their consent were excluded from the study.

### 2.2. Study Design

Clinical data: During a routine consultation, the rheumatologist gave the patient a document providing all the explanations relating to the clinical investigation. After having obtained his/her informed consent, the investigator collected in the medical file demographic data (age, gender, weight, body mass index—BMI), co-morbidities and related treatments, current and past treatments for knee OA, number of previous VSs, IA corticosteroid injections, IA steroid injections (past and/or concomitant with VS), and level of activity (sedentary, active, athletic).

Radiological data: Knee radiographs were centrally read by the same experienced investigator (TC) for assessment of the Kellgren–Lawrence grades [37] and the involved compartments (patellofemoral (PF), tibiofemoral (TF) or both).

Treatment outcomes: For his/her part, the patient had to fill out a questionnaire including a self-assessment of the duration of treatment effectiveness (DoE) measured as the number of weeks during which VS was effective on symptoms, and the level of satisfaction with the treatment on OA symptoms assessed semi-quantitatively (very satisfied, satisfied, little satisfied, dissatisfied) as well as the level of satisfaction with DoE (satisfied, dissatisfied).

### 2.3. Statistical Analysis

Qualitative variables were described using frequencies. Quantitative variables were described using mean values, standard deviation (SD), and characteristics of their distribution (minimum, maximum, and median). Univariate analysis was performed using the chi-square test, Fischer’s exact test, or the Mann–Whitney test, as appropriate. A multivariate regression analysis pooled factors with a significant association at the 0.2 threshold in previous bivariate regressions. All the analyses were carried out using R++© software1.4 (Zebrys SAS, Toulouse, France), with the alpha threshold at 0.05.

## 3. Results

Eighty-nine patients (124 knees) were included in the analysis. The thirty-three males and fifty-six females had a median age of 67 (range 22–90) years and a median BMI of 26.6 kg/m^2^ (range 18.3–42.8). Fifty subjects were retired, nine were unemployed and thirty were still working. Forty-one patients practiced sport regularly. Sixty patients were taking one or more medications for osteoarthritis. A total of 81 knees (65.3%) had a synovial fluid effusion at the time of viscosupplementation (mean volume 1.9 ± 4.8 mL). Eight patients received IA triamcinolone hexacetonide (2 mL/40 mg) concomitantly to HA injection. The main characteristics of patients are given in Table 1.

**Figure 1:**
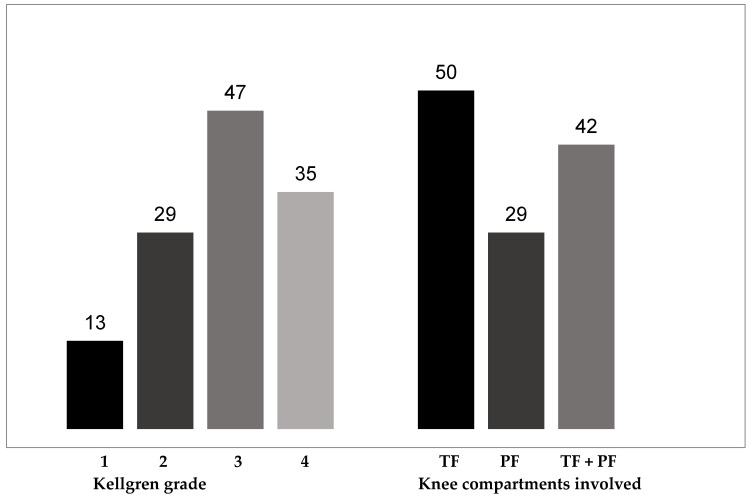
Radiological characteristics of the knees at the time of hyaluronic acid injection (n = 124).

Among the 124 knees treated, the K–L score was I, II, III, and IV in 13, 29, 47, and 35 cases respectively. The joint space narrowing affected the following knee compartment(s) TF, PF and both in 50, 29, and 42 knees respectively (three missing data). Radiological features at entry are summarized in Figure 1. The average (SD) DoE was 50.5 ± 24.7 weeks (16–156 weeks). A very high majority of patients reported that they were satisfied with the treatment (very satisfied 49.2%, satisfied 39.5%). Three-quarters of the patients said they were satisfied with the DoE of treatment (very satisfied 38.7%, satisfied 37.1%). The average DoE was 51.3 ± 25.9 weeks (17–156 weeks) in satisfied patients and 44.0 ± 22.3 weeks (16–84 weeks) in those who were dissatisfied (*p* = 0.039).

In the bivariate analysis, satisfaction with VS was unrelated to gender (*p* = 0.77), bilaterality (*p* = 0.61), disease duration (*p* = 0.61), analgesics consumption at time of injection (*p* = 0.95), sport practice (*p* = 0.90), presence of a synovial fluid effusion at time of injection as well as its volume (*p* = 0.20), and treatment tolerability (*p* = 0.99). There was a trend of a lower rate of satisfaction in subjects with at least one comorbidity (X^2^ = 3.32; *p* = 0.09).

Satisfaction with treatment was highly correlated to patient’s satisfaction with DoE (Fisher exact test; *p* < 0.00001), but surprisingly only moderately correlated with DoE in the weeks of efficacy (Fisher exact test; *p* = 0.024). The proportion of satisfied patients was higher in patients with BMI < 27.5 kg/m^2^ (OR 3.15 [95CI 1.01–10.1]; *p* = 0.045). The median BMI of satisfied patients was 26. 5 mg/m^2^ (range 18.3–42.8) and that of dissatisfied ones was 27.8 (range 21.2–35.1). The rate of satisfaction was significantly higher in active versus sedentary patients (Fisher exact test; *p* = 0.007), in tibiofemoral unicompartmental involvement (Fisher exact test; *p* = 0.005), in K–L grade 1 to 3 versus 4 (Fisher exact test; *p* = 0.01) and in subjects who did not require a corticosteroid injection at the time of the HA injection (OR 4.68 [95CI 1.02–21.4]; *p* = 0.02). The main results of the bivariate analysis are given in Table 2.

Compared to dissatisfied patients, satisfied subjects were older (66.5 ± 12.7 versus 58.7 ± 15.3 years; *p* < 0.001) and were more frequently seeking a new VS treatment at the time of consultation (X^2^ = 10.9; *p* = 0.006).

In the multivariate analysis, only four items were found to be associated with a higher rate of satisfaction: older age (*p* = 0.0004), K–L grade < 4 (*p* = 0.017), absence of corticosteroid injection (*p* = 0.02), and DoE (*p* = 0.04). The multivariate analysis data are given in Table 3.

## 4. Discussion

This study shows that a single IA injection of HANOX-M-XL provides long-lasting pain relief over one year in a large majority of patients with mild to moderate knee OA. These findings confirm the preliminary results reported by Perruchet et al. [33], who found a mean (SD) duration of efficacy of 52 (24.7) weeks in 51 patients with knee OA. It is not surprising that patients with a higher BMI and more advanced radiological OA were less likely to be satisfied with a HA IA injection. Obesity and radiological severity have repeatedly been shown to be strong and independent predictors of poor prognosis after knee VS [24,35,38]. However, as we have previously shown [39], obesity is not a formal contra-indication to VS since some obese patients benefit durably from IA HA. The radiological severity of OA has been repeatedly shown to be one of the most important predictors of poorer response to VS [24,33,36,38,39,40]. Our cohort is no exception to the rule, showing a greater rate of satisfaction in patients with K–L grade 1 to 3 versus 4. However, it must be highlighted that, among the 35 patients with K–L4, only 10 (28.5%) were not satisfied with the treatment, showing that VS can be effective even in advanced radiographic stages. Unicompartmental tibiofemoral involvement was also found to be associated with a higher rate of satisfaction. In patients with patellofemoral OA, the presence of altered pain processing and sensitization with increased sensitivity have been evidenced [41,42]. Patellofemoral syndrome is a condition that is difficult to treat, in which fear-avoidance beliefs are the main predictor of rehabilitation outcome [43,44]. Although patellar cartilage lesions are among the most frequent pathologies found in knee arthroscopy [45], no HA versus placebo-controlled trial has been published. However, the EUROVISCO recommendations mentioned that patellofemoral involvement may negatively influence the response to viscosupplementation in patients with tibiofemoral OA, with a moderate level of agreement [26]. The rate of satisfaction was also higher in active versus sedentary patients, once again confirming the importance of physical activity in the management of OA [46,47]. Surprisingly the concomitant IA corticosteroid injection with HA was shown to be associated with a lower rate of satisfaction. Several prospective studies [48,49] have demonstrated that the combination of HA and corticosteroids improves the short-term effect of viscosupplementation and has no positive or negative effect on long-term effectiveness. In our study, patients who received both HA and corticosteroids were likely those who were most symptomatic and needed rapid pain relief.

Interestingly, satisfied patients were significantly older than those who were little or not satisfied. Age remained a predictor of satisfaction in the multivariate analysis independent of the K–L grade. We can hypothesize that younger subjects are more demanding about the result because they want to lead a normal life without any limitations, whereas older people more easily accept limiting their activities because of their knee. However, Altman et al. showed that a decrease in pain and function scores after VS was unrelated to patient age [24].

These discrepancies between clinical results and satisfaction suggest that being satisfied with a treatment is not only related to the improvement of symptoms but also to other factors, probably dependent on the expectations of the patient. Power et al. [50] reported that while patients with diabetes reported similarly high rates of satisfaction with total hip and knee replacement as patients without diabetes, diabetes had a negative impact on improvements after surgery, as evidenced by the fact that diabetic patients were less likely to meet clinically important improvement criteria. On the contrary, patients may be disappointed if the result obtained is not the expected one. This is probably the reason why several patients in our cohort were dissatisfied with the DoE of the treatment, even though this duration was much longer than that of some very satisfied patients (i.e., 84 weeks). Conversely, other patients in whom the benefit was only short-lived (i.e., 17 weeks) still declared themselves satisfied with the treatment. It has been shown that, beyond the clinical benefit, there are several factors that can positively or negatively influence satisfaction with treatments. Factors which are most clearly related to satisfaction include accessibility to medical care, duration of treatment, perceived expertise and personality of physicians, the clarity of care-givers’ communication, patient expectations, socio-demographic characteristics, and health status of the patient [51]. We did not record the patients’ beliefs and expectation before HA injection, and we cannot judge the clarity of our explanations. However, before taking the decision on VS, which is always taken by mutual agreement with the patient, we give detailed information on the treatment, the risk–benefit ratio, and the chance of success according to the findings of Eymard et al. [38]. At the end of the consultation, we hand the patient a booklet, provided by the manufacturer, summarizing the essential information regarding the treatment. Since all the patients in our cohort received the same information, we are unable to draw conclusions about the influence of the patient information on the clinical outcome.

Obviously, our study has strengths and limitations. The main strength is that it was conducted in daily practice. Patients were treated under routine conditions and were only made aware of the trial when they returned to hospital. Unlike in prospective clinical trials, there were no exclusion criteria linked to age, OA severity or BMI. Our cohort was quite representative of the population usually treated. Without questioning the indispensable nature of randomized controlled trials (RCTs), the latter can be criticized for their “generalizability” issues; in other words, how well trial participants represent those who receive the treatments in daily clinical practice [52]. In making treatment decisions, physicians must consider the relevance of the results of clinical trials that are closely dependent on external validity, which is, unfortunately, often poor. This can lead to underuse of treatments that are, however, effective [52]. A retrospective analysis of RCT data to compare trial participants’ socio-demographic characteristics, clinical features, and health outcomes to a representative sample of U.S. adults with chronic spinal pain showed that populations adversely affected by health disparity were not well represented in these clinical trials [53]. Migliore et al. [22] highlighted the discrepancy between recommendations, based on meta-analyses of randomized-controlled trials, and clinical practice for viscosupplementation. To be as close as possible to daily practice conditions, we used patient satisfaction as the main judgment criterion because, in a real situation, the patient’s opinion is the main factor in evaluating the effectiveness of viscosupplementation. Therefore, it is very likely that our results are a true reflection of reality.

Obviously, the study suffers from several limitations. The main limitation lies in the recruitment method. After VS, we usually advise patients to return as soon as the pain returns. However, an appointment is systematically scheduled 1 year after the injection, in order to systematically evaluate the treated knee(s), even in completely asymptomatic subjects. In patients at high risk of progression or very symptomatic at the time of the injection, an appointment is scheduled 6 months after the injection. However, we cannot quantify the number of patients who did not return and who chose another treatment option because they did not obtain sufficient pain relief. On the other hand, some completely asymptomatic patients have probably not returned either and it is not possible for us to count them. Another limitation is that the study was focused on patients treated with HANOX-M-XL, making it impossible to extrapolate the current results to other HA products [54] and alternative injection protocols (i.e., non-cross-linked HA and a repeated injection scheme).

## 5. Conclusions

In this study conducted in real-life conditions, we identified several predictive factors of patient satisfaction after viscosupplementation of the knee with a single injection of HANOX-M-XL. Alongside objective factors (BMI, age, radiological stage, extent of osteoarthritis, concomitant injection of a corticosteroid), there are probably subjective factors, more difficult to highlight because they are linked to patient beliefs, fears and expectations, impacting their satisfaction. A study specifically considering these subjective factors would provide a better understanding of the disparities in clinical response to viscosupplementation.

## Figures and Tables

**Table 1:** Patients characteristics at time of hyaluronic acid injection (n = 89).

**Gender/n (%)**	
Female	56 (63%)
Male	33 (37%)
**Treated knees/n (%)**	
One	54 (60.7%)
Two	35 (39.3%)
**Age (Years)**	
Median	67
Mean ± SD (range)	66.2 ± 12.8 (22–90)
**Body mass index (kg/m^2^)**	
Median	26.6
Mean ± SD (range)	26.9 ± 5.2 (18.3–42.8)
**Professional status/n (%)**	
Working	30 (33.7%)
Unemployed	9 (10.1%)
Retired	50 (56.2%)
**Sport practice/n (%)**	30 (33.7%)
Yes N (%)	41 (46.1%)
No N (%)	48 (53.9%)
**Patients taking drugs for knee OA/n (%)**	60 (67.4%)
Analgesics	36 (40.4%)
NSAIDs	9 (10.1%)
SYSADOAs	23 (25.8%)
**Corticosteroid injection/n (%)**	8 (9%)

Abbreviations: SD: standard deviation; NSAIDS: non-steroidal anti-inflammatory drugs; SYSADOAs: symptomatic slow acting drugs for osteoarthritis.

**Table 2:** Comparison between satisfied and dissatisfied patients treated with a single injection of HANOX-M-XL for knee osteoarthritis.

Predictor	Satisfied	Dissatisfied	*p*
**Age: Median (range)** Years 89 patients	68.5 (22–90)	61.7 (41–84)	0.006
**BMI: Median (range)** kg/m^2^ 89 patients	26.5 (18.3–42.8)	27.8 (21.2–35.1)	0.045
**Active n (%)** **Sedentary n (%)** 89 patients	63 (81%) 6 (54.5)	15 (19) 5 (45.4)	0.007
**KL 1–3 n (%)** **KL 4 n (%)** 124 knees	80 (89.9) 30 (85.7)	9 (10.1) 5 (14.3)	0.01
**Isolated TF n (%)** **PF or TF + PF n (%)** 124 knees	52 (92.5) 56 (82.4)	4 (7.5) 12 (17.6)	0.005
**Corticosteroid injection** **Yes n (%)** **No n (%)** **124 knees**	6 (66.7) 103 (90.3)	3 (33) 11 (9.7)	0.02

Abbreviations: BMI: body mass index; KL: Kellgren–Lawrence radiological grade; TF: tibiofemoral involvement; PF: patello-femoral involvement.

**Table 3:** Predictors of satisfaction with viscosupplementation with a single injection of HANOX-M-XL for knee osteoarthritis: multivariate analysis (ANOVA).

Predictors	F Value	*p*-Value
Age	145.3	0.0004
KL < 4	102.9	0.017
Absence of IACS	34.3	0.02
DoE	24.2	0.04

Abbreviations: KL: Kellgren–Lawrence radiological grade; IACS: intra-articular corticosteroid; DoE: duration of effectiveness.

## Data Availability

Data and materials are available at the Clinical Research Unit of the Nord Franche=Comté Hospital, Trévenans, France.
